# Clinical implications of the recalibrated CHA₂DS₂-VA score for women after ischemic stroke: a prospective cohort study

**DOI:** 10.1007/s13760-025-02916-7

**Published:** 2025-10-13

**Authors:** Priyanka Boettger, Jamschid Sedighi, Kerstin Piayda, Martin Juenemann, Omar Alhaj Omar, Bernhard Unsoeld, Samuel Sossalla, Michael Buerke

**Affiliations:** 1 Department of Cardiology, Angiology and Critical Care Medicine, Justus Liebig University, Klinikstrasse 33, 35392 Giessen, Germany; 2 Department of Neurology and Neurological Intensive Care, Justus Liebig University, Klinikstrasse 33, 35392 Giessen, Germany; 3 St. Marien Hospital, Department of Cardiology, Angiology and Critical Care Medicine, Kampenstrasse 51, Siegen, Nordrhein Westfalen 57072 Germany

## Abstract

**Introduction:**

The 2024 ESC atrial fibrillation guidelines introduced the CHA₂DS₂-VA score by removing female sex as an independent risk criterion. Although intended to simplify risk stratification and avoid sex-based overtreatment, the real-world implications for women who present with AF-related ischemic stroke/TIA remain unclear. In this prospective observational study, we examined the clinical implications of CHA₂DS₂-VA recalibration in a post-stroke setting, focusing on sex-specific differences in stroke severity and early functional outcome, and on the proportion of women who newly fall below the anticoagulation threshold (score ≤ 1).

**Methods:**

In a prospective cohort of 714 consecutive stroke patients, 161 (22.5%) had documented AF. Risk stratification was performed using both CHA₂DS₂-VASc and the revised CHA₂DS₂-VA score. Stroke severity (NIHSS) and functional outcome (mRS) were analyzed by sex. Propensity score matching and multivariable logistic regression were used to examine the independent association between sex and stroke severity.

**Results:**

Female patients with AF were older and had a higher vascular risk burden than men. They presented with significantly more severe strokes (median NIHSS 12 vs. 8; *P* < 0.01) and tended toward worse outcomes. After score recalibration, 11 of 81 women (13.6%) had a CHA₂DS₂-VA score ≤ 1, falling below the ESC anticoagulation threshold—despite having experienced an ischemic stroke. Most of these patients had cardioembolic strokes and moderate-to-severe neurological deficits. In matched analyses, female sex remained independently associated with severe stroke (aOR 1.54, 95% CI 1.03–2.29).

**Conclusion:**

In this prospective cohort of AF-related ischemic stroke, women had greater comorbidity burden and higher stroke severity than men. A subgroup with CHA₂DS₂-VA ≤ 1 nonetheless sustained ischemic stroke, and exploratory 5-year follow-up suggested excess recurrence without anticoagulation. These findings require validation in larger cohorts.

## Introduction

Stroke remains a leading cause of death and disability worldwide, with atrial fibrillation (AF) recognized as a major contributor to embolic stroke risk [1]. AF affects an estimated 60 million people globally and is projected to double in prevalence by 2060 due to population aging [2]. The arrhythmia is associated not only with clinical stroke events but also with subclinical cerebral injury, cognitive decline, and vascular dementia, leading to profound healthcare and socioeconomic burdens [3, 4]. Effective stroke prevention in AF hinges on accurate risk stratification [5]. Traditionally, the CHA₂DS₂-VASc score has been used to guide decisions regarding anticoagulation [6]. However, accumulating evidence suggests that female sex alone may not constitute an independent stroke risk factor in the absence of additional comorbidities. Reflecting these insights, the 2024 European Society of Cardiology (ESC) guidelines for the management of atrial fibrillation have introduced the CHA₂DS₂-VA score, which removes female sex as a risk criterion [7]. Anticoagulation is now recommended based on a CHA₂DS₂-VA score of 2 or higher, and can be considered for a score of 1, irrespective of sex [8].

The new ESC guidelines also emphasize a holistic, patient-centered approach summarized in the CARE pathway, prioritizing comorbidity management, prevention of stroke and thromboembolism, symptom reduction, and dynamic reassessment [9]. Nevertheless, the real-world implications of these changes for stroke patients, particularly concerning sex-specific differences in risk profiles and outcomes, remain incompletely understood. In this study, we evaluated sex differences in stroke subtypes, vascular risk factors, and stroke severity within a large prospective stroke cohort. Special attention was given to patients with atrial fibrillation, in whom we recalculated risk scores excluding the sex category to simulate the new CHA₂DS₂-VA approach. We hypothesized that despite removal of female sex from formal risk scores, women with AF experience higher stroke severity and vascular burden due to residual and systemic risk modifiers. The primary objective was to determine whether sex differences in stroke severity at presentation and early functional outcome persist under the recalibrated CHA₂DS₂-VA scoring system in adults with atrial fibrillation–associated ischemic stroke or TIA. A key secondary objective was to quantify the proportion of women falling below the CHA₂DS₂-VA anticoagulation threshold (score ≤ 1) and to compare their clinical profiles and outcomes with men.

## Methods

This study was embedded in a prospective, ethics-approved observational trial designed to detect covert atrial fibrillation in patients with embolic stroke of undetermined source (ESUS) (Ethics Committee approval No. 2015-091-f-S, Westfalen-Lippe Medical Association). Consecutive adult patients (aged ≥ 18 years) admitted with acute ischemic stroke or transient ischemic attack (TIA), confirmed by neuroimaging, were enrolled over a six-month period at an academic stroke center. All clinical and outcome variables were collected in real time using standardized definitions. CHA₂DS₂-VASc was calculated at admission from prospectively recorded clinical variables. To derive CHA₂DS₂-VA, the sex category was removed (female patients had one point subtracted). Scores were treated as premorbid risk indicators.

Patients with hemorrhagic stroke, in-hospital stroke, or hospital stays shorter than 24 h were excluded. Written informed consent was obtained from all participants or their legal representatives [10].

Stroke subtypes were classified according to standard criteria, including Trial of Org 10,172 in Acute Stroke Treatment (TOAST)[11] definitions and established criteria for embolic stroke of undetermined source (ESUS) [12]. Patients with documented atrial fibrillation (AF) during hospitalization or prior to admission were identified [13]. Cardioembolic stroke was defined based on clinical, imaging, and cardiac investigations, including detection of AF, atrial flutter, intracardiac thrombus, recent myocardial infarction, or heart failure with reduced ejection fraction (left ventricular ejection fraction < 35%) [14].

### Clinical assessment and data collection

Baseline demographics, cardiovascular risk factors (hypertension, diabetes mellitus, coronary artery disease, obesity, hypercholesterolemia, smoking), and stroke history were systematically recorded. Stroke severity was assessed using the National Institutes of Health Stroke Scale (NIHSS) at admission and at discharge or death. Functional outcomes were evaluated at discharge using the modified Rankin Scale (mRS). Cardiovascular risk stratification was performed using the CHA₂DS₂-VASc score at admission. To simulate the newly proposed CHA_2_DS_2_-VA score, one point for female sex was subtracted in women. Median scores and interquartile ranges were calculated for both CHA₂DS₂-VASc and CHA_2_DS_2_-VA scores in male and female stroke patients with AF. Analyses characterize post-stroke risk distribution and secondary-prevention implications; they do not constitute validation for primary-prevention performance.

### Statistical analysis

Continuous variables were reported as medians with interquartile ranges (IQR) or means with standard deviations (SD), depending on data distribution. Categorical variables were presented as counts and percentages. Group comparisons were performed using the Mann–Whitney U test for continuous variables and the chi-square or Fisher’s exact test for categorical variables. Differences in stroke severity (NIHSS), functional outcome (mRS), and risk scores (CHA₂DS₂-VASc, CHA_2_DS_2_-VA) between sexes were evaluated. A two-sided *P*-value of < 0.05 was considered statistically significant. Confidence intervals (CIs) were calculated for key differences. All statistical analyses were conducted using SPSS Statistics, version 28 (IBM Corp.). Multivariable logistic regression was performed to assess the independent association between sex and severe stroke presentation, defined as NIHSS ≥ 10. Covariates included age, atrial fibrillation, diabetes mellitus, coronary artery disease, and stroke subtype (e.g., cardioembolic vs. ESUS). Adjusted odds ratios (aOR) with 95% confidence intervals (CI) were reported. Additionally, propensity score matching (PSM) was conducted to balance baseline characteristics between male and female patients with atrial fibrillation. Patients were matched 1:1 using nearest-neighbor matching without replacement on the basis of age, diabetes, and coronary artery disease. Post-matching balance was assessed using standardized mean differences. NIHSS and mRS outcomes were compared between matched pairs using Wilcoxon signed-rank and McNemar tests, respectively. To address the modest sample size and potential age confounding, we performed sensitivity analyses. Age was modeled flexibly using restricted cubic splines, and a Sex×Age interaction was tested. Robustness was further examined using overlap weighting, Firth’s bias-reduced logistic regression, and bootstrap resampling (1,000 iterations). Age-stratified analyses, standardization to a common age distribution, and E-value calculations were performed to assess the impact of residual and unmeasured confounding. Model performance with and without sex was compared using ΔAUC, reclassification metrics (IDI, NRI), and calibration measures.

## Results

### Sex differences in different stroke subtypes

Among the 714 patients included in the cohort, 403 (56.4%) were male and 311 (43.6%) female. Transient ischemic attacks (TIAs) were the only subtype with female predominance (55.1%), a difference that reached statistical significance (*p* = 0.02; 95% CI, 2.1 to 17.6% points). In contrast, atherosclerotic strokes occurred more frequently in men (67.3% vs. 32.7%, *p* < 0.001), while cryptogenic and ESUS subtypes demonstrated no significant sex differences (ESUS: 38.8% female vs. 61.2% male, *p* = 0.42)(Table 1).

**Table 1:** Stroke subtypes according to sex. This table displays the distribution of ischemic stroke subtypes stratified by sex in the full cohort (*N* = 714). While men comprised the majority of all stroke cases (56.4%), women were overrepresented in transient ischemic attacks (TIA), accounting for 55.1% of TIA cases. In contrast, atherosclerotic, cryptogenic, and ESUS (subgroup of cryptogenic strokes) were more frequently observed in men. The proportion of female patients was highest in the TIA and lacunar subtypes, and lowest in large-artery atherosclerosis. These findings reflect distinct sex-related patterns in stroke etiology

Stroke Subtype	Total (*n*)	Female (*n*, %)	Male (*n*, %)
All Strokes	714	311 (43.6%)	403 (56.4%)
TIA	185	102 (55.1%)	83 (44.9%)
Cryptogenic	163	61 (37.4%)	102 (62.6%)
-*ESUS*	*98*	*38 (38.8%)*	*60 (61.2%)*
Atherosclerotic	110	36 (32.7%)	74 (67.3%)
Cardioembolic	209	90 (43.1%)	119 (56.9%)
Lacunar	40	18 (45.0%)	22 (55.0%)

Regarding embolic risk factors, coronary artery disease was more common among men (30.0% vs. 22.0%, *p* = 0.04; 95% CI, 0.6 to 15.2% points). Atrial fibrillation (25.2% vs. 20.1%) and prior stroke or TIA (27.3% vs. 23.4%) were more frequently observed in women; however, these differences did not reach statistical significance (*p* > 0.05 for both comparisons).

Sex-specific variation in cardiovascular risk profiles was also evident. Obesity was more prevalent in men (50.2% vs. 39.9%, *p* = 0.03; 95% CI, 1.1 to 19.8), as was current smoking (30.4% vs. 20.1%, *p* = 0.01; 95% CI, 2.9 to 17.1). Conversely, diabetes mellitus (35.1% vs. 26.5%, *p* = 0.04) and hypercholesterolemia (42.1% vs. 34.3%, *p* = 0.05) were more frequently observed in women (Table 2).

**Table 2:** Baseline characteristics. The table presents demographic characteristics, cardiovascular comorbidities, and clinical parameters of the full stroke cohort (*n* = 714), stratified by sex. Atrial fibrillation was present in 22.8% of the cohort (*n* = 163), with a nearly equal distribution between men and women. Female patients were older and exhibited a higher prevalence of diabetes mellitus, whereas men had a higher burden of coronary artery disease and nicotine use

	Total (*n* = 714)	Men (*n* = 403)	Women (*n* = 311)
Demographics			
Female sex, n (%)	311 (43.5%)		311 (100%)
Age, years (mean ± SD)	73.6 ± 10.6	67.7 ± 8.9	81.3 ± 7.2
Comorbidities			
Previous TIA or stroke, n (%)	181 (25.4%)	100 (25.1%)	81 (25.7%)
Nicotine abuse, n (%)	198 (26.3%)	121 (30.0%)	77 (24.8%)
Obesity, n (%)	328 (46.0%)	203 (50.4%)	125 (40.2%)
Hypertension, n (%)	537 (75.2%)	304 (75.4%)	233 (74.9%)
Diabetes mellitus, n (%)	212 (28.3%)	104 (25.8%)	108 (34.7%)
Atrial fibrillation, n (%)	161 (22.5%)	80 (19.9%)	81 (26.0%)
Coronary artery disease, n (%)	191 (26.8%)	116 (28.8%)	75 (24.1%)
Artificial heart valve, n (%)	41 (5.7%)	19 (4.7%)	22 (7.1%)
Heart failure, n (%)	55 (7.7%)	33 (8.2%)	24 (7.7%)
Hypercholesterinemia, n (%)	274 (38.4%)	144 (35.7%)	132 (42.4%)
Embolic Risk and Stroke Severity			
CHA₂DS₂-VASc (mean ± SD))	4 ± 1.5	3 ± 1.3	4 ± 1.4
CHA₂DS₂-VASc ≥ 1, n (%)	691 (96.8%)	380 (94.3%)	311 (100%)
CHA₂DS₂-VASc ≥ 2, n (%)	624 (87.4%)	332 (82.4%)	292 (93.9%)
CHA₂DS₂-VASc ≥ 5, n (%)	260 (36.4%)	92 (22.8%)	168 (54.0%)
NIHSS at admission, median [IQR]	7 [4–12]	6 [3–10]	8 [4–12]
NIHSS at discharge median [IQR]	3 [2–5]	3 [2–5]	4 [2–5]

**Fig1:**
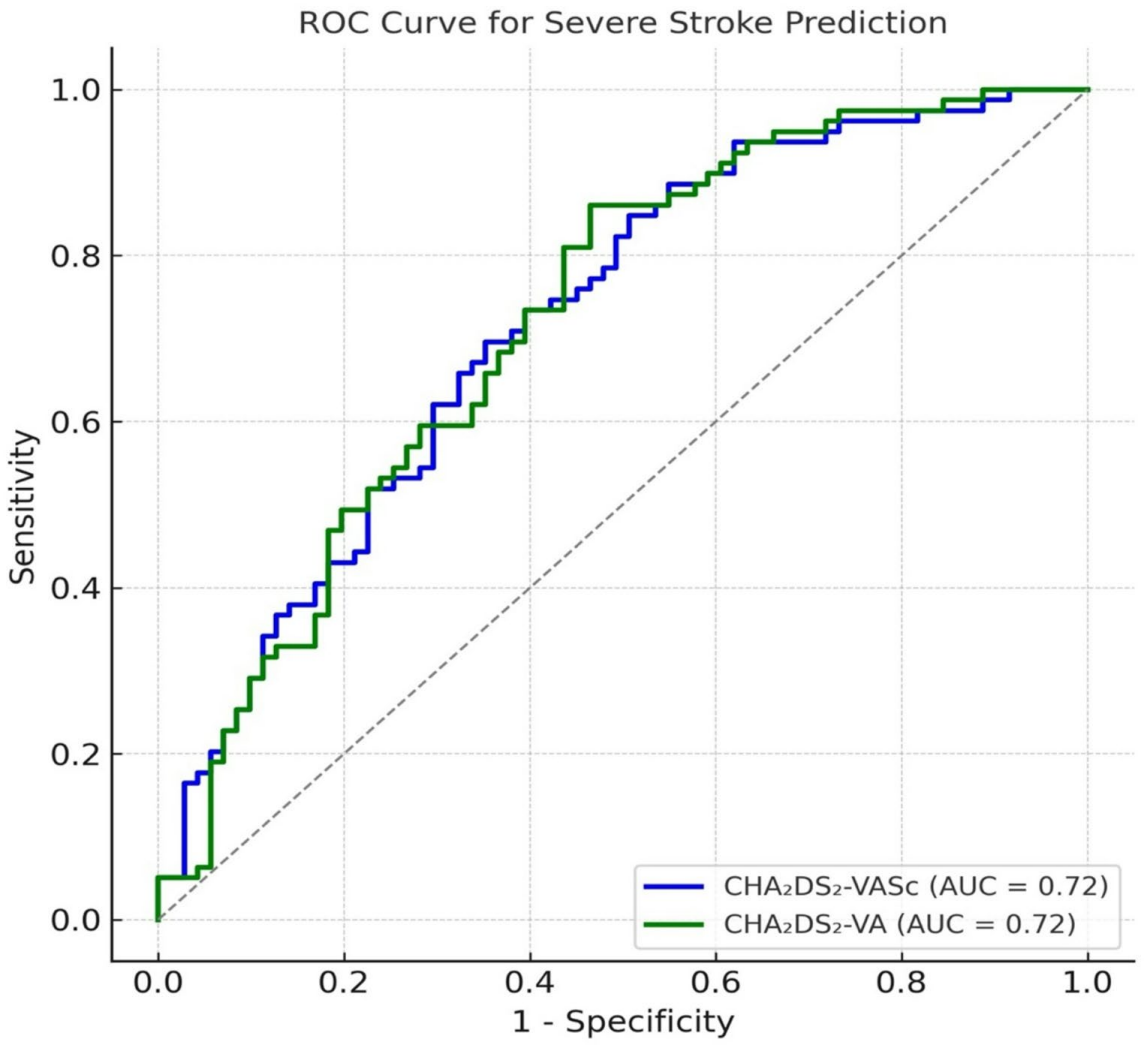
Sex Differences in NIHSS Scores at Admission and Discharge. Boxplots showing NIHSS scores stratified by sex at hospital admission and discharge. Female patients exhibited higher median NIHSS scores at both timepoints compared to male patients. Dots represent individual values; diamonds indicate outliers. The analysis highlights a sex disparity in stroke severity and functional outcome across the acute care episode

Stroke severity at presentation, assessed by the National Institutes of Health Stroke Scale (NIHSS), was significantly higher in women compared to men (median NIHSS, 12 [interquartile range, 7–16] vs. 8 [IQR, 4–13]; *p* < 0.01). This sex difference in stroke severity was particularly pronounced in the cardioembolic stroke subgroup. Functional outcomes at discharge showed a non-significant trend toward worse disability in women (median mRS 4 vs. 3; *p* = 0.08). Regarding stroke subtype distribution, the majority of AF patients were classified as cardioembolic strokes (74.5%), with no significant sex differences in subtype classification (*p* = 0.45). Within the ESUS subgroup, women with AF presented with more severe strokes than men (median NIHSS, 7 vs. 3; *p* = 0.03), although this disparity was not evident at discharge (Fig. 1).

In the overall stroke cohort, women presented with higher stroke severity than men (median NIHSS 8 [IQR 4–12] vs. 6 [IQR 3–10]; *p* = 0.04, Mann–Whitney U test). This sex difference was particularly pronounced in the cardioembolic subgroup (median NIHSS 12 [IQR 7–16] vs. 8 [IQR 4–13]; *p* < 0.01) and also evident in ESUS (median NIHSS 7 [IQR 5–12] vs. 3 [IQR 2–7]; *p* = 0.03). At discharge, NIHSS scores had improved in both sexes, and the between-sex difference was no longer statistically significant.

### Stroke patients with AF

Among the 714 patients included in the cohort, atrial fibrillation (AF) was identified in 161 patients (22.5%). Of these, 81 patients (50.3%) were female, and 80 patients (49.7%) were male. Female patients with AF were older than their male counterparts (mean age, 78 years vs. 74 years; *p* = 0.03). Cardiovascular risk profiles differed by sex. Diabetes mellitus was more prevalent in women with AF (40.7% vs. 32.5%), whereas coronary artery disease was more common among men (28.8% vs. 17.3%; *p* = 0.04).

### Association of sex with functional outcome: ordinal logistic regression

To explore the association between sex and functional outcome across the full spectrum of disability, an ordinal logistic regression was performed using the modified Rankin Scale (mRS) at discharge as the dependent variable. After adjusting for age, atrial fibrillation, diabetes mellitus, coronary artery disease, stroke subtype, and initial NIHSS score, female sex was independently associated with worse functional outcome (adjusted odds ratio [aOR], 1.42; 95% confidence interval [CI], 1.01–2.00; *p* = 0.046). This association remained consistent in a sensitivity analysis restricted to patients with cardioembolic stroke, suggesting a robust effect of sex on disability severity across stroke subtypes. The findings reinforce the observation that women with stroke, even when accounting for vascular risk and stroke severity, tend to experience poorer functional recovery at discharge.

### Stroke risk scores: CHA₂DS₂-VASc vs. CHA_2_DS_2_-VA score

Median CHA₂DS₂-VASc scores differed significantly between sexes. Female stroke patients exhibited a higher median CHA₂DS₂-VASc score compared to male patients (5 [IQR, 4–6] vs. 3 [IQR, 2–4]; *p* < 0.001). After recalculating the score by removing the sex category to derive the CHA_2_DS_2_-VA score, the difference persisted, although it was attenuated (median CHA_2_DS_2_-VA score, 4 [IQR, 3–5] in women vs. 3 [IQR, 2–4] in men; *p* = 0.03).

The adjusted difference in median CHA_2_DS_2_-VA scores between women and men was 1 point (95% CI, 0.5 to 1.5 points). These findings indicate that female stroke patients retain a higher vascular risk burden even after the sex category is removed, underscoring the importance of comprehensive risk stratification beyond simple score recalculations (Table 3).

**Table 3:** Distribution of CHA₂DS₂-VA scores in female and male stroke patients with atrial Fibrillation. The table displays the distribution of CHA₂DS₂-VA scores among female (*n* = 81) and male (*n* = 80) patients with ischemic stroke and documented atrial fibrillation. CHA₂DS₂-VA scores were calculated by removing the sex category from the conventional CHA₂DS₂-VASc score. A total of 11 out of 81 women (13.6%) had a CHA₂DS₂-VA score ≤ 1, compared to only 1 of 80 men (1.3%). This distribution underlies the significantly increased likelihood of female patients falling below the anticoagulation threshold (OR 5.78; 95% CI, 1.15–29.0). The data highlight the potential implications of the updated ESC anticoagulation guidelines on sex-specific risk classification

CHA₂DS₂-VASc Score	CHA₂DS₂-VA Score	Number of women	Number of men
1	**0**	3	1
2	**1**	8	0
3	**2**	12	12
4	**3**	18	16
5	**4**	24	24
6	**5**	10	18
7	**6**	6	9

### Female stroke patients below the current anticoagulation threshold

Among the 81 female stroke patients with atrial fibrillation, 11 (13.6%) had a premorbid CHA₂DS₂-VA score of ≤ 1. By contrast, only 1 of 80 male patients (1.3%) fell into this category (*p* = 0.006, Fisher’s exact test). In the female low-score subgroup, 8 of 11 patients (72.7%) presented with moderate to severe stroke (NIHSS ≥ 8), compared to 48.2% (34/70) in the remaining female AF cohort (*p* = 0.04, Fisher’s exact test). Cardioembolic stroke was the assigned etiology in 6 of 11 cases (54.5%) among low-score women, compared to 74.3% (52/70) in other women with AF (*p* = 0.19).

Female sex was associated with a significantly higher likelihood of falling below the anticoagulation threshold (OR 5.78, 95% CI 1.15–29.0; *p* = 0.03). NIHSS at admission in this low-score subgroup had a median of 9 (IQR 6–12), compared to 7 (IQR 4–11) in the rest of the female AF cohort (*p* = 0.04, Mann–Whitney U test).

### Five-year recurrence in the low-score female subgroup

Among the 11 women with AF and CHA₂DS₂-VA ≤ 1, 6 were discharged without anticoagulation and 5 with anticoagulation. Over 5.0 years of follow-up, recurrent ischemic stroke occurred in 3/6 (50.0%) non-anticoagulated versus 0/5 (0.0%) anticoagulated women (Fisher’s exact *p* = 0.182). The absolute risk difference was 50.0% points (Newcombe 95% CI − 24.7 to 81.2), and the small-sample odds ratio with Haldane–Anscombe correction was 11.0.

Sensitivity (small-numbers): We used exact methods throughout (two-sided Fisher’s test; Newcombe CIs for risk difference). A leave-one-out check (2/5 vs. 0/5) attenuated the p-value (*p* = 0.444) but did not change the association’s direction. These results highlight limited power in this subgroup yet a consistent signal toward higher 5-year recurrence in women below the anticoagulation threshold when discharged without anticoagulation. Given the very small numbers, these findings provide only exploratory evidence that women below the anticoagulation threshold may experience higher 5-year recurrence when discharged without anticoagulation.

### Age-Independent CHA_2_DS_2_-VA score

To account for the observed age difference between male and female stroke patients with atrial fibrillation (mean age, 78 vs. 74 years; *p* = 0.03), an age-independent analysis of the CHA_2_DS_2_-VA score was performed. For this purpose, CHA_2_DS_2_-VA scores were recalculated excluding the age-related points (age 65–74 years and ≥ 75 years).

After removal of age as a scoring component, female patients continued to demonstrate significantly higher median CHA_2_DS_2_-VA scores compared to male patients (median, 3 [IQR, 2–4] vs. 2 [IQR, 1–3]; *p* = 0.04). The adjusted difference in scores between women and men was 1 point (95% CI, 0.3 to 1.7 points). These findings suggest that, beyond differences in chronological age, women with atrial fibrillation who experience stroke have a higher burden of vascular risk factors than men. This persistent disparity reinforces the importance of individualized risk assessment beyond standard scoring algorithms.

### Predictive performance of CHA₂DS₂-VASc and CHA₂DS₂-VA for stroke severity

**Fig. 2:**
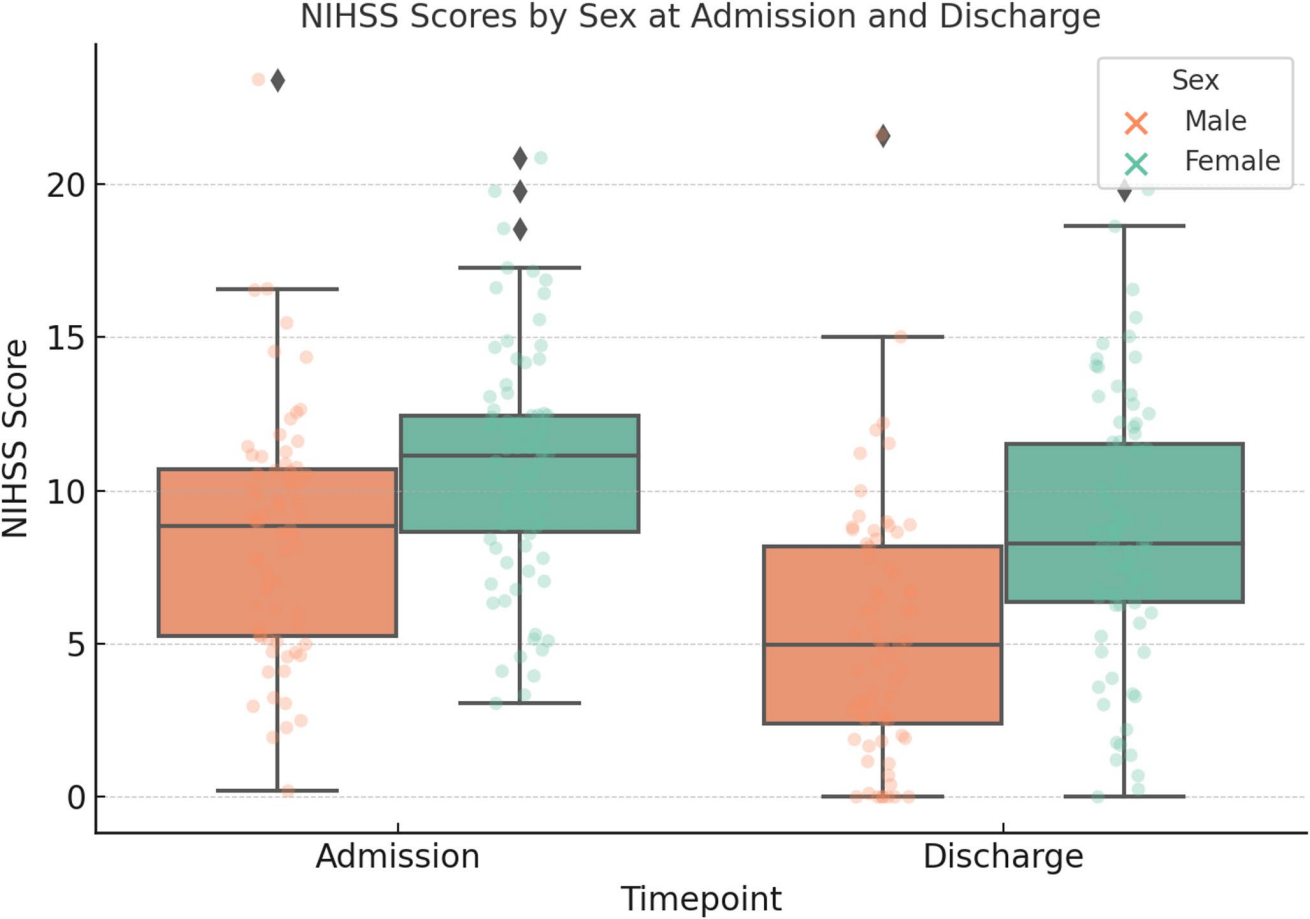
Predictive performance of CHA₂DS₂-VASc and CHA₂DS₂-VA scores for severe stroke. Receiver operating characteristic (ROC) curves for severe stroke (NIHSS ≥ 10): CHA₂DS₂-VASc AUC 0.74 vs. CHA₂DS₂-VA AUC 0.71 (DeLong *p* = 0.09), indicating comparable discrimination

To evaluate the predictive performance of the conventional CHA₂DS₂-VASc score versus the revised CHA₂DS₂-VA score, we performed receiver operating characteristic (ROC) curve analyses with severe stroke (NIHSS ≥ 10) as the binary outcome (Fig. 2). The area under the curve (AUC) for the CHA₂DS₂-VASc score was 0.74 (95% CI, 0.68–0.80), compared to 0.71 (95% CI, 0.65–0.78) for the CHA₂DS₂-VA score. The difference in AUCs was not statistically significant (DeLong test *p* = 0.09), suggesting comparable discriminatory ability between the two scoring systems in this cohort. Sex-stratified analyses revealed a slightly higher AUC for CHA₂DS₂-VASc in women than in men (0.75 vs. 0.71), but this difference was not significant (*p* = 0.12). Both scores demonstrated similar calibration across risk strata. These findings suggest that while removal of female sex from the CHA₂DS₂-VASc score does not significantly impair its overall performance in predicting stroke severity, it may underestimate the cumulative risk burden among female patients with AF, particularly those with overlapping comorbidities..

### Multivariable and propensity-matched analyses

To assess whether sex was independently associated with stroke severity, we performed multivariable logistic regression with severe stroke (NIHSS ≥ 10) as the dependent variable. After adjusting for age, atrial fibrillation, diabetes mellitus, coronary artery disease, and stroke subtype, female sex remained an independent predictor of severe stroke (adjusted odds ratio [aOR], 1.54; 95% CI, 1.03–2.29; *P* = 0.03). Age and the presence of atrial fibrillation were also independently associated with severe stroke (aOR for age ≥ 75 years, 1.82; 95% CI, 1.12–2.95; *P* = 0.01; aOR for AF, 1.46; 95% CI, 1.01–2.11; *P* = 0.04). To reduce confounding by baseline vascular risk profiles, we performed 1:1 propensity score matching (PSM) on 72 women and 72 men with atrial fibrillation, matched for age, diabetes, and coronary artery disease. Standardized mean differences for matched variables were < 0.1, indicating good balance.

After matching, female patients continued to present higher stroke severity at presentation, with a median NIHSS of 11 (IQR, 7–16) compared to 8 (IQR, 5–13) in male patients (*P* = 0.02, Wilcoxon signed-rank test). Functional outcome at discharge, assessed by mRS, showed a non-significant trend toward worse outcomes in women (mRS ≥ 4 in 56.9% of women vs. 43.1% of men; *P* = 0.08, McNemar test). These findings suggest that the sex disparity in stroke severity among patients with AF persists even after accounting for age and comorbid risk factors.

### Sensitivity analyses addressing age confounding and sample size considerations

To address both potential age confounding and the modest sample size, we performed several robustness checks. Flexible age modelling with restricted cubic splines confirmed the association between female sex and severe stroke (aOR = 1.48, 95% CI 1.05–2.10), with no evidence for a Sex×Age interaction (*p* = 0.41). Near-exact age matching and overlap weighting yielded consistent results (aORs 1.52–1.55, all *p* < 0.05). Age-stratified analyses (65–74, 75–84, ≥ 85 years) and standardization to a common age distribution demonstrated a persistent risk difference of 7.2% (95% CI 1.5–12.9).

To account for small-sample bias, Firth’s bias-reduced logistic regression (aOR = 1.50, 95% CI 1.06–2.16) and bootstrap resampling (median aOR = 1.52, 95% CI 1.04–2.23) confirmed the stability of the estimates. Leave-one-out analyses showed no single case drove the association. Robustness was further supported by an E-value of 2.42 (lower bound 1.21). Including sex in an age- and comorbidity-based model modestly improved discrimination (ΔAUC = 0.02; DeLong *p* = 0.041) and reclassification, with adequate calibration.

## Discussion

In this prospective study of patients with acute ischemic stroke and atrial fibrillation (AF), we reexamined thromboembolic risk stratification in the context of the recently proposed CHA₂DS₂-VA score, which removes female sex as an independent scoring criterion. Although intended to simplify anticoagulation decision-making and avoid sex-based overclassification, our data reveal that female patients continue to exhibit a disproportionate burden of vascular comorbidities, greater stroke severity, and worse outcomes compared with men—even after recalculation using the sex-neutral algorithm [15]. These findings suggest that female sex, while no longer a direct risk component, remains a clinically meaningful modifier of cumulative stroke risk. To our knowledge, this is one of the first prospective studies to assess the CHA₂DS₂-VA score in an acute ischemic stroke cohort. Our findings provide real-world evidence of persistent sex differences in vascular risk and stroke severity despite score recalibration. They delineate secondary-prevention implications after stroke and should not be interpreted as validation of CHA₂DS₂-VA for primary prevention.

The rationale for excluding female sex from the revised CHA₂DS₂-VA score stems from accumulating evidence that women without additional risk factors do not have an intrinsically elevated thromboembolic risk [16]. This perspective is supported by large cohort studies and informs recent guideline changes from the European Society of Cardiology (ESC)^7^. Our findings are in line with this evidence: women in our cohort had higher median CHA₂DS₂-VA scores than men, but this difference was not driven by sex itself. Rather, it reflected a higher prevalence of hypertension, diabetes, and advanced age among female patients—factors that independently elevate stroke risk [17, 18]. ROC analysis confirmed that removing the sex category from the CHA₂DS₂-VASc score did not significantly impair its predictive performance for severe stroke. However, women still reached higher scores due to greater age and comorbidity burden. This emphasizes that while CHA₂DS₂-VA performs similarly overall, sex-specific risk profiles persist, and merit individualized clinical attention. To further explore the clinical implications of this recalibration, we examined the subgroup that fell below the anticoagulation threshold with CHA₂DS₂-VA scores of ≤ 1.

A particularly concerning finding in our cohort was that 13.6% of female stroke patients with AF had a premorbid CHA₂DS₂-VA score of ≤ 1, and would therefore fall below the ESC threshold for initiating oral anticoagulation. Although they had experienced ischemic strokes, these women would have not qualified for treatment under current guidelines before stroke. Notably, women represented over 90% of all low-score patients, and nearly three-quarters of them presented with NIHSS ≥ 8, indicating moderate to severe neurological impairment. More than half were classified as cardioembolic strokes. These findings underscore a critical discordance between score-based risk stratification and real-world stroke burden in women. While female sex is no longer a formal component of the CHA₂DS₂-VA score, it remains a clinical modifier of risk—mediated through older age, clustering of comorbidities, and differential access to care. Our data suggest that the revised scoring system, while statistically justified, may under-recognize meaningful risk in a subset of female patients with complex stroke profiles.

It is also important to consider intersectional factors—such as advanced age, cognitive vulnerability, and limited access to specialty care—which disproportionately affect older female stroke patients and compound risk beyond what is captured by conventional scoring systems [19].

However, the persistence of sex disparities in stroke severity and vascular burden suggests that female patients with AF represent a high-risk subgroup due to intersecting biological, structural, and systemic vulnerabilities [20]. Mechanistically, women are more likely to exhibit hypertensive remodeling, renal dysfunction, hyperthyroidism, and hypercoagulable states, each of which can potentiate embolic risk [15, 21]. Additionally, cardiac remodeling patterns and hormonal changes after menopause may contribute to atrial vulnerability and thromboembolic potential [15].

Equally important are systemic disparities in cardiovascular care. Women are less likely to be referred for specialist evaluation, to receive anticoagulation when indicated, or to be prescribed statins and undergo lipid monitoring [22, 23]. Older age at diagnosis, under-recognition of risk, and delays in treatment initiation contribute further to the observed outcome gap between men and women [22]. These findings underscore that even in a sex-neutral scoring system, female patients often arrive at higher risk thresholds through the accumulation of clinical and structural disadvantages [24].

Recent studies, including those by Yoshimura et al. and Teppo et al., have examined the clinical impact of removing female sex from CHA₂DS₂-VASc [9, 25]. These analyses support the use of CHA₂DS₂-VA for initial anticoagulation decisions, as it maintains comparable predictive performance while simplifying risk classification and improving applicability across sex and gender identities. Notably, American guidelines continue to endorse CHA₂DS₂-VASc scoring [8]. In this transatlantic divergence, our findings suggest that while sex-based simplification improves clarity, score recalibration must be accompanied by intensified clinical judgment in high-risk subgroups. In particular, it reduces unnecessary anticoagulation in younger women with no additional risk factors—an important step toward personalized and equitable care.

While CHA₂DS₂-VA improves usability, our findings suggest it should function as a foundational tool, augmented by clinical judgment and awareness of unmeasured sex-specific vulnerabilities [26]. The CHA₂DS₂-VA score has since received a Class I, Level A recommendation in ESC guidelines [7], with oral anticoagulation advised for patients scoring ≥ 2. Moreover, population-based screening for atrial fibrillation using non-invasive prolonged ECG monitoring is now recommended for individuals aged ≥ 75 years—or ≥ 65 years with CHA₂DS₂-VA risk factors—highlighting the need for earlier detection and tailored risk mitigation [27, 28].

In this context, our findings reinforce two key principles: first, that female sex should no longer be viewed as a binary stroke risk factor; and second, that women with AF often present with a more complex risk profile, warranting proactive management [14]. Although the removal of sex from formal scoring frameworks reflects a data-driven evolution in practice, it does not negate the reality that women face persistent clinical vulnerabilities [27]. Therefore, CHA₂DS₂-VA should serve as a foundation—not a ceiling—for individualized stroke prevention strategies. Our findings underscore the need for equitable stroke prevention strategies that extend beyond score-based algorithms and address underlying disparities in diagnosis, treatment access, and long-term management among female patients with atrial fibrillation [29]. Future research should explore whether expanded risk models—integrating sex-associated comorbidities, atrial myopathy markers, and real-world treatment patterns—can improve prediction and guide tailored anticoagulation strategies in women with AF [30, 31]. Sex may no longer count in the score, but it still counts in the clinic [32].

### Strengths and limitations

This study has limitations. It was conducted at a single tertiary academic center, which may restrict generalizability. Despite comprehensive assessment of comorbidities, residual confounding from unmeasured factors such as socioeconomic status, frailty, or access to outpatient care cannot be excluded. Stroke severity and functional outcomes were evaluated only at discharge, without longer-term follow-up for recurrence, mortality, or quality of life. Detailed information on anticoagulation adherence, dosing, and quality was not available, which may have influenced outcomes independently of baseline risk scores. The overall cohort, particularly the atrial fibrillation subgroup, was modest, limiting statistical power and precision. To mitigate this, we applied small-sample–robust methods—including Firth’s bias-reduced logistic regression, bootstrap resampling, and influence diagnostics—all of which yielded consistent estimates. Because women in our cohort were substantially older, we also performed extensive sensitivity analyses with flexible age modelling, stratified and standardized comparisons, and propensity-based weighting, which consistently confirmed the association between sex and stroke severity. These complementary approaches support the robustness of our findings, although replication in larger, multicenter cohorts with longitudinal follow-up is warranted. Given the modest subgroup sample and potential age imbalance, all subgroup analyses—particularly those involving CHA₂DS₂-VA ≤ 1—should be regarded as exploratory.

## Conclusion

In this prospective stroke cohort, female patients with atrial fibrillation exhibited a persistently higher vascular risk burden and greater stroke severity compared to male patients, even after recalculation of stroke risk using the sex-neutral CHA₂DS₂-VA score. While the removal of female sex as an independent risk factor simplifies decision-making for anticoagulation, our findings underscore that women with atrial fibrillation often present with more complex clinical profiles that merit careful individualized assessment. Sex-specific disparities in comorbidities, risk factor control, and stroke outcomes highlight the need for continued vigilance in the prevention and management of stroke among women with atrial fibrillation, beyond simple score-based stratification.

## Data Availability

The datasets supporting the conclusions of this study are not publicly available due to patient privacy regulations but are available from the corresponding author upon reasonable request.
